# Intravenous Levosimendan versus Inhalational Milrinone in the Management of Pulmonary Hypertension during Adult Cardiac Surgery: A Randomized Clinical Trial

**DOI:** 10.3390/life14091164

**Published:** 2024-09-14

**Authors:** Panagiotis Ftikos, Georgios Gkantinas, Vlasios Karageorgos, Anna Smirli, Nektarios Kogerakis, Evangelos Leontiadis, Konstantinos Petsios, Theofani Antoniou, Kassiani Theodoraki

**Affiliations:** 1 Department of Anesthesiology, Onassis Cardiac Surgery Center, 176 74 Athens, Greece; pftikos@yahoo.gr (P.F.); gadinasgio@gmail.com (G.G.); bkarageorgos@hotmail.com (V.K.); an.smirli@gmail.com (A.S.); antoniou_fani@yahoo.gr (T.A.); 2 Department of Cardiac Surgery, Onassis Cardiac Surgery Center, 176 74 Athens, Greece; nkoger@gmail.com; 3 Department of Cardiology, Onassis Cardiac Surgery Center, 176 74 Athens, Greece; evanleont@gmail.com; 4 Faculty of Nursing, National and Kapodistrian University of Athens, 115 28 Athens, Greece; cpetsios@nurs.uoa.gr; 5 Department of Anesthesiology, Aretaieion University Hospital, National and Kapodistrian University of Athens, 115 28 Athens, Greece

**Keywords:** levosimendan, milrinone, pulmonary hypertension, cardiac surgery, right ventricular dysfunction, cardiopulmonary bypass

## Abstract

**Introduction:** The perioperative management of patients with pulmonary hypertension (PH) undergoing cardiac surgery is challenging, mainly due to the potential risk of right ventricular failure (RVF). Levosimendan is a calcium-sensitizing agent that has primarily been used in the treatment of decompensated heart failure. However, recently levosimendan has been shown to be an effective and safe therapeutic strategy for patients with pulmonary arterial hypertension and PH associated with left heart disease. The aim of this study was to investigate the potential utility of the preemptive administration of levosimendan in cardiac surgical patients with preexisting PH and to compare its effectiveness with milrinone, which represents an already established therapeutic option in the management of PH during cardiac surgery. **Materials and Methods:** In this study, 40 adult cardiac surgical patients with PH were randomly assigned to receive either levosimendan intravenously or milrinone via inhalation in a double-blind fashion prior to a cardiopulmonary bypass (CPB). Hemodynamic and echocardiographic parameters were recorded and evaluated before and after the administration of the drugs. **Results and Conclusions:** The results of this study demonstrated that both levosimendan and milrinone administered before CPB in cardiac surgical patients with PH may offer protective benefits, reducing pulmonary artery pressure and preventing the exacerbation of PH and RVF. Pulmonary vasodilation attributed to levosimendan is of longer duration and greater magnitude compared to pulmonary vasodilation afforded by milrinone.

## 1. Introduction

Pulmonary hypertension (PH) is an important prognostic factor in cardiac surgery associated with increased morbidity and mortality [1,2]. This fact primarily hinges on the relation existing between PH and right ventricular failure (RVF), which can severely complicate cardiac surgery, requiring early recognition and diligent perioperative management [3,4,5].

PH is a broad term that describes a wide spectrum of disease states in which elevated pulmonary artery pressure and pulmonary vascular resistance (PVR) promote right ventricular (RV) dysfunction, eventually leading to RVF [6]. The clinical classification of PH distinguishes five groups: (1) pulmonary arterial hypertension (PAH); (2) PH caused by left heart disease; (3) PH caused by lung disease or hypoxia; (4) chronic thromboembolic PH; and (5) PH due to unclear or multifactorial mechanisms [7]. PH in its various forms affects ≈1% of the global population, and up to 10% of individuals >65 years of age [8]. The most frequent type of PH in patients presenting for cardiac surgery is PH resulting from left heart disease. However, with advancing average age and the rising prevalence of comorbidities among cardiac surgical patients in recent years, it is increasingly probable that various forms of PH will be encountered within this patient group [9,10].

Irrespective of the underlying cause, PH may be exacerbated during cardiac surgery due to several causes. The most important factors contributing to this include systemic inflammatory response, ischemia–reperfusion injury and endothelial damage after cardiopulmonary bypass (CPB) [11,12,13], the administration of protamine [14,15], transfusion of blood products [16,17] and prothesis mismatch after heart valve surgery [18,19]. The exacerbation of preexisting PH during cardiac surgery causes increased RV afterload and has the potential to result in RVF. In fact, the most significant consequence of uncontrolled PH is the development of RVF, which, in turn, is accompanied by decreased pulmonary blood flow, decreased left ventricular preload, decreased cardiac output (CO) and arterial blood pressure. The subsequent decline in coronary blood flow further deteriorates RV function, establishing a destructive vicious cycle that ultimately leads to supra-systemic pressures in the right ventricle and eventual cardiovascular collapse. In the perioperative context, RVF is related to mortality rates ranging from 22% to 90%, along with notable complications such as an elevated requirement for inotropic and mechanical circulatory support, extended periods of mechanical ventilation, prolonged stay in the intensive care unit (ICU) and prolonged hospital duration of stay [20].

Diagnosing and managing RVF in the perioperative setting is a challenging task. Diagnosis is complicated and treatment poses even greater demands. Intraoperatively, it often manifests as challenges in weaning off CPB, while it can manifest as low CO syndrome or dysfunction in multiple organs during the postoperative period. Currently, no established evidence-based guidelines exist for managing RVF in cardiac surgical patients with PH during the perioperative period. The primary goal of management is to prevent the onset of RVF. This is achieved through strategies aimed at decreasing RV afterload, optimizing preload and enhancing the contractility of the right ventricle [21,22,23]. In this context, numerous pharmacologic agents administered intravenously or via inhalation have been used in order to prevent the deterioration of PH and to manage RVF during cardiac surgery. These agents include prostaglandins, nitric oxide, milrinone, adrenergic agents and levosimendan [24,25,26].

Milrinone is a phosphodiesterase inhibitor with positive inotropic and vasodilatory effects. Its mechanism of action involves increasing intracellular levels of cyclic adenosine monophosphate by inhibiting the enzyme phosphodiesterase type 3. Milrinone is one of the most widely used drugs in cardiac surgery for PH management. A notable drawback of the intravenous administration of milrinone is systemic hypotension. In order to avoid this complication, an alternative approach is to administer milrinone through inhalation. Inhaled milrinone administered before CPB reduces the severity of PH during cardiac surgery and has the potential to facilitate weaning from CPB in patients with PH [27,28,29,30].

Levosimendan is a calcium-sensitizing agent with inotropic, vasodilatory and cardioprotective properties [31]. Levosimendan improves myocardial contractility by increasing the affinity of myocardial troponin C to calcium. Unlike other inotropic agents, levosimendan exerts a positive inotropic effect without leading to calcium overload or an elevation in myocardial oxygen demand. Additionally, levosimendan displays vasodilatory effects by the opening of adenosine triphosphate (ATP)-dependent K+ channels in vascular smooth muscle cells, causing vasodilation in both arterial and venous smooth muscle cells. The cardioprotective properties of levosimendan are associated with the opening of mitochondrial ATP-dependent K+ channels in the cardiomyocytes, providing protection against ischemia–reperfusion injury. Finally, levosimendan exerts anti-inflammatory, antiapoptotic and antioxidative effects [32]. Levosimendan has a fast onset of action and a short half-life of approximately one hour. However, it can have a long duration of action primarily due to the creation of an active metabolite known as OR-1896 [33]. Considering the vasodilatory properties of levosimendan, it can expand the pulmonary vasculature as well as the venous system, leading to a decrease in both RV preload and afterload. Additionally, due to its inotropic effects, levosimendan can potentially enhance RV contractility [34,35]. Recently, levosimendan has been shown to be an effective and safe therapeutic strategy for patients with PAH and PH due to left heart disease [36]. However, despite the potential benefits of levosimendan in managing PH and related RV failure, there are limited available data regarding its application in the context of cardiac surgery.

The aim of the study was to examine the pharmacodynamic effect of levosimendan in patients with preexisting PH undergoing cardiac surgery with the use of CPB, evaluate the efficacy of its intravenous administration prior to CPB in preventing the exacerbation of PH and RVF, and compare its use with the inhalational administration of milrinone.

## 2. Materials and Methods

### 2.1. Study Population

This prospective, randomized, double-blind, single-center interventional study received approval from the Ethics Committee of the Onassis Cardiac Surgery Center under reference number 701/08.12.2020. Prior to the surgery, informed consent was obtained from every patient. The study was registered on clinicaltrials.org with the identifier NCT04718350 before patient enrollment.

The study population consisted of 40 patients with severe PH due to left heart disease, undergoing elective cardiac surgery. Patients were considered to have severe PH if systolic pulmonary artery pressure (SPAP) was greater than 55 mmHg or mean pulmonary arterial pressure (MPAP) was greater than 25 mmHg. These pressure values were determined using preoperative transthoracic or transesophageal echocardiography (TEE) or right heart catheterization. Inclusion criteria were adult patients with PH caused by left heart disease, undergoing elective cardiac surgery with the use of extracorporeal circulation. Exclusion criteria, used in order to rule out conditions that could confound the study, were left ventricular ejection fraction (LVEF) <30%, severe renal failure, hepatic failure, acute or chronic thromboembolic disease, and chronic obstructive pulmonary disease.

### 2.2. Patient Randomization

Patients were randomly assigned into two treatment groups: Group A, receiving levosimendan at a dose of 6 mcg/kg intravenously; and Group B, receiving milrinone at a dose of 50 mcg/kg via inhalation. Randomization was performed by using a computer-generated random code.

### 2.3. Intraoperative Anesthetic Protocol

Consistency was ensured in premedication, monitoring, anesthesia and mechanical ventilation. In addition to standard monitoring for cardiac anesthesia, a pulmonary artery catheter (Swan-Ganz) was used in order to confirm the presence of PH and a comprehensive TEE exam was performed in all patients in order to obtain detailed information about cardiac function. Anesthetic induction in all patients was performed with intravenous doses of midazolam 0.05 mg/kg, fentanyl 2 mcg/kg and rocuronium bromide 1 mg/kg, while for the maintenance of anesthesia, all patients received sevoflurane at an end-tidal concentration of 0.5% to 2% and intravenous doses of midazolam and fentanyl every hour, targeting the bispectral index (BIS) values of 40 to 50. Concerning mechanical ventilation, a protective ventilation strategy with tidal volumes of 6 mL/kg and positive end-expiratory pressure (PEEP) of 5 cm H_2_O was employed in all patients, aiming at normocapnia (PCO_2_ 35–40 mmHg). Blood transfusion practice aimed at maintaining hemoglobin concentration between 9 and 10 g/dL.

### 2.4. Intraoperative Hemodynamic Management

All perioperative hemodynamics parameters and TEE findings were evaluated by a Swan-Ganz catheter (7.5F, Edwards LifeScience, Irvine, CA, USA) and a Vivid 3 echocardiography device (General Electric, Hamburg, Germany). The variables measured or calculated included heart rate (HR), systolic arterial pressure (SAP), diastolic arterial pressure (DAP), mean arterial pressure (MAP), SPAP, diastolic pulmonary artery pressure (DPAP), MPAP, mean pulmonary to mean arterial pressure ratio (MPAP/MAP), central venous pressure (CVP), pulmonary capillary wedge pressure (PCWP), stroke volume (SV), CO, cardiac index (CI), systemic vascular resistance (SVR), systemic vascular resistance index (SVRI), PVR, pulmonary vascular resistance index (PVRI), tricuspid annular plane systolic excursion (TAPSE) and LVEF. CO was assessed using the thermodilution technique with three injections of room-temperature 5% dextrose 10 mL, and PCWP was measured at end-expiration. CO, SV, SVR and PVR were indexed to body surface area (BSA), calculated by using the Du Bois formula (BSA = 0.007184 × (Height (m)^0.725^) × (Weight (kg)^0.425^).

Hemodynamics were treated according to the following protocol during and after surgery: (1) CVP or PCWP at values between 10 to 12 mmHg and 16 to 20 mmHg, respectively, with fluid administration; (2) MAP at values between 60 and 90 mmHg with norepinephrine 0.05 mcg/kg/min increased incrementally by 0.02 mcg/kg/min until the MAP was 60 mmHg; (3) if CI < 2 L/min/m^2^, inotropic support was started initially with dobutamine 2–10 mcg/kg/min, followed (if necessary) by the addition of epinephrine at 0.01–0.1 mcg/kg/min.

### 2.5. Transesophageal Echocardiographic Parameters

All TEE exams were performed by anesthesiologists certified in adult TEE by the European Association of Cardiovascular Imaging and reviewed offline by a cardiologist blinded to the allocation group. According to the American Society of Echocardiography guidelines, left ventricular systolic function was assessed by calculating the left ventricle ejection fraction (LVEF) using the modified Simpson method, while right ventricular function was evaluated by measuring tricuspid annular plane systolic excursion (TAPSE) [37].

### 2.6. ICU Management

Patients were extubated once they were adequately warmed, hemodynamically stable, and with normal arterial blood gas levels. ICU discharge criteria included the following: SpO_2_ > 90% at FiO_2_ of 0.5 by facemask, hemodynamic stability without intravenous inotropic or vasopressor support, urine output > 0.5 mL/kg/h, and chest tube drainage < 50 mL/h.

### 2.7. Pharmacological Treatment

Eligible patients were randomized to receive either a single dose of inhaled milrinone or levosimendan intravenously. The study drug was administered after the induction of anesthesia, confirmation of PH presence and once the baseline hemodynamic profile and TEE exam had been completed.

Inhaled milrinone was administered through a jet nebulizer device for aerozol generation (Aeroneb Pro Micropump Nebulizer, Aerogen Ltd., Galway, Ireland) attached to the ventilatory circuit, near the endotracheal tube. The concentration of milrinone used in the inhaled milrinone group was 1 mg mL^−1^ and the cumulative dose administered was 50 mcg kg^−1^. Levosimendan diluted in 100 mL (dextrose 5% water) was administered as a continuous infusion at a dose of 6 mcg/kg. The duration of administration for both agents was 20 min approximately. Per protocol, drug administration was discontinued in the event of an anaphylactic reaction, refractory hypotension (defined as a MAP < 60 mmHg despite optimal therapeutic management) or intractable arrhythmias and the patient was excluded from the protocol and further analysis.

In order to ensure blindness, patients of group A, apart from levosimendan, received N/S 0.9% via inhalation, while patients of group B, except milrinone, were also administered a mixed-vitamin solution with a yellow color, devoid of relevant cardiovascular effects and indistinguishable in appearance and volume from levosimendan.

Before and after the study drug administration, intraoperative hemodynamic and transesophageal echocardiographic parameters were evaluated via Swan-Ganz catheter and TEE and recorded in our database. Hemodynamic measurements and transesophageal echocardiographic evaluation were performed after the induction of anesthesia and before drug administration (baseline), 20 min after discontinuation of CPB, at the end of surgery and two hours after ICU admission. The additional parameters assessed included the need for further inotropic or vasopressor support during weaning from CPB, the requirement for intra-aortic balloon pump (IABP) or extracorporeal membrane oxygenation (ECMO) and the duration of mechanical ventilation, as well as the length of ICU and hospital stay.

### 2.8. Study Outcomes

The primary outcome of the study was the expected change in MPAP values and MPAP/MAP ratio after levosimendan administration and the comparison with the respective changes afforded by milrinone. We postulated that a decrease in MPAP with simultaneous sparing of the systemic circulation by levosimendan administration, a fact already shown with milrinone inhalation in the context of cardiac surgery, would be suggestive of levosimendan-mediated selective pulmonary vasodilation. Based on the available literature, additional outcomes were expected PVR changes which, along with echocardiographic evaluation, could provide additional proof of the favorable effect of intravenous levosimendan on pulmonary vasculature and cardiac function.

## 3. Statistical Analysis

Sample size calculation was based on power analysis methodology with a design of two groups of the between-subject factor (levosimendan and milrinone) and four levels of the within-subject factor of time. For this design, 20 participants per study group achieved a power of 0.85 for the between-subject main effect at an effect size of 0.40; a power of 0.95 for the within-subject main effect at an effect size of 0.24; and a power of 0.95 for the interaction effect at an effect size of 0.24. Quantitative variables were tested for normality of distribution via the Kolmogorov–Smirnov criterion. Quantitative variables were expressed as mean values (standard deviation) and as the median (interquartile range), while categorical variables were expressed as absolute numbers and frequencies. For the comparison of proportions, the Chi-square and Fisher’s exact tests were used. Student’s *t*-tests or Mann–Whitney tests were used for the comparison of continuous variables between the two groups, as appropriate. A repeated-measures analysis of variance (ANOVA) was used to evaluate the changes observed in parameters between the two groups over the follow up period. Bonferroni correction was used in the case of multiple testing in order to control for type I error. All reported *p*-values are two-tailed. Statistical significance was set at *p* < 0.05 and analyses were conducted using the SPSS statistical software (version 26.0).

## 4. Results

In total, 46 patients were screened for enrollment in the study. Of those, 40 patients fulfilled the inclusion criteria and completed the study. Therefore, the sample consisted of 40 patients, divided in two equally sized groups. Discontinuation of drug administration due to untoward reactions was not necessary for any patient. One group received levosimendan intravenously (group A) and the other received milrinone via inhalation (group B). Their characteristics are presented in Table 1, by group. No significant differences were found between the two groups as far as their characteristics were concerned. No statistically significant differences were found between the two groups in the requirement for vasopressor or inotropic support during weaning from CPB, in the requirement for IABP or ECMO use, in the duration of mechanical ventilation during ICU stay, in the length of ICU and hospital stay, and in mortality during hospitalization.

HR, SAP, DAP, MAP, CVP, SPAP, DPAP, MPAP and MPAP/MAP values at various timepoints by group and changes in HR, SAP, DAP, MAP, CVP, SPAP, DPAP, MPAP and MPAP/MAP over time are presented in Table 2 and Table 3, respectively.

At baseline and throughout follow-up, HR was similar in both groups. HR was significantly increased in group A 20 min after CPB discontinuation compared to the baseline (*p* = 0.009), and thereafter, there were no significant changes between consecutive measurement HR values in either group. The degree of change was similar in the two groups.

SAP was similar in the two groups at baseline and throughout the follow-up period. There were no significant changes between consecutive SAP measurements in group B, while in group A, there was a significant increase from the measurement made at the end of surgery to the measurement made two hours after ICU admission (*p* = 0.019). However, the degree of change over time was similar in the two groups.

Furthermore, DAP was similar in the two groups at baseline and throughout the follow-up period. There were no significant changes between consecutive DAP measurements in any of the groups. The degree of change over time was similar in the two groups.

MAP was similar in the two groups at baseline, as well as throughout the follow-up time. There were no significant changes between consecutive measurements in MAP values in any of the groups. The degree of change over time was similar in the two groups.

Additionally, CVP values were similar between the two groups at baseline, 20 min after the discontinuation of CPB and two hours after ICU admission. However, at the end of surgery, the CVP values of group A were significantly greater than those of group B (*p* = 0.05). Timewise, in group A, there was a significant decrease from the measurement made at the end of the surgery to the measurement made two hours later (*p* = 0.006). In group B, there was no significant change throughout the follow-up period. The degree of change over time tended to differ between groups, with group A having a later decrease than group B (*p* = 0.088).

**Figure 1:**
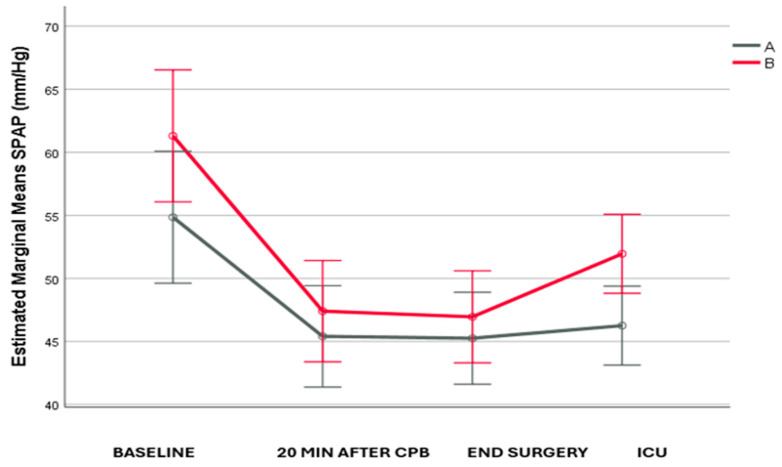
SPAP changes across the follow-up period.

SPAP values were similar between the two groups at baseline, 20 min after the end of CPB and at the end of the surgery. However, two hours after ICU admission, SPAP in group A was lower than in group B (*p* = 0.013). Regarding the changes over time, it was found that there was a significant decrease in both groups 20 min after weaning from CPB compared to the baseline (*p* = 0.003 and *p* < 0.001, respectively). In group A, there was no significant further change, while in group B, there was a significant increase two hours after ICU admission compared to the end of surgery (*p* = 0.014) (Figure 1). The degree of change was similar in the two groups.

DPAP was similar between the two groups at baseline, 20 min after the end of CPB and at the end of the surgery. However, two hours post-surgery, DPAP in group A was lower than in group B (*p* < 0.001). Regarding the changes over time, it was found that in group B there was a significant decrease 20 min after the termination of CPB compared to baseline (*p* = 0.017), and a significant increase from the end of surgery until two hours later (*p* = 0.022). In group A, no significant change was found. The degree of change differed significantly between groups, as B had significant changes while group A did not (*p* = 0.008).

MPAP was similar between the two groups initially, 20 min after weaning from CPB and at the end of the surgery. However, two hours post-surgery, MPAP in group A was lower than in group B (*p* < 0.001). Regarding changes over time, it was found that there was a significant decrease in both groups 20 min after CPB discontinuation compared to the initial measurements (*p* = 0.001 and *p* < 0.001, respectively). In group A, there was no further significant change, while in group B, there was a significant increase two hours after surgery compared to the end of surgery (*p* = 0.004). The degree of change differed significantly between the two groups, with group A maintaining a decrease and group B having an increase in MPAP values at the end of follow-up (*p* = 0.02).

**Figure 2:**
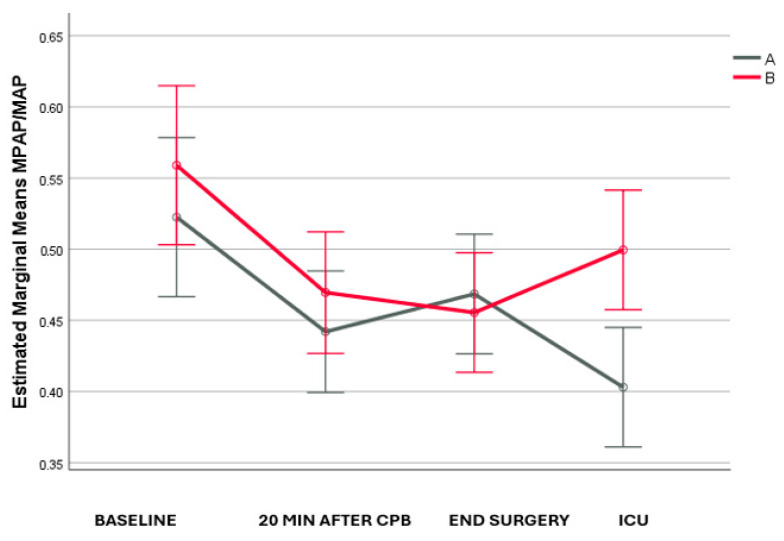
MPAP/MAP changes across the follow-up period.

MPAP/MAP (Figure 2) values were similar between the two groups at baseline, 20 min after CPB discontinuation, as well as at the end of the surgery. However, two hours after surgery, MPAP/MAP in group A was lower than in group B (*p* = 0.002). Over time, in both groups, there was a significant decrease from baseline until 20 min after CPB discontinuation (*p* = 0.040 and *p* = 0.017, respectively). Furthermore, in group A, there was a significant decrease from the end of the surgery until two hours later (*p* = 0.008). The degree of change over time differed significantly between groups, with group A having a greater overall decline than group B (*p* = 0.017).

The PCWP, CO, CI, SV, SVI, SVR, SVRI, PVR and PVRI values at various timepoints by group and changes in PCWP, CO, CI, SV, SVI, SVR, SVRI, PVR and PVRI over time are presented in Table 4 and Table 5, respectively.

At baseline, no significant differences were found between the two groups. PCWP, CO, CI, SV, SVI, SVR and SVRI were similar between the two groups throughout follow-up at all timepoints. Regarding changes over time, there were no significant differences between consecutive PCWP, SV and SVI measurements in either group, but as far as CO and CI are concerned, a significant increase was shown in both groups 20 min after the discontinuation of CPB compared to the initial measurements (*p* = 0.005 and *p* = 0.055 for CO for the two groups, respectively, and *p* = 0.006 and *p* = 0.034 for CI for the two groups, respectively). Afterward, there were no further significant changes in any of the groups. The degree of PCWP, CO, CI, SV, SVI, SVRI, PVR and PVRI changes over time was similar in the two groups.

Regarding SVR, in group B, no significant differences were found between consecutive measurements, while in group A, there was a significant decrease 20 min after CPB discontinuation compared to baseline (*p* = 0.001) and a significant increase two hours post-surgery compared to the end of the surgery (*p* = 0.001). The degree of SVR change over time tended to differ between the two groups, as group A had significant changes while group B did not (*p* = 0.095).

Similarly, in group A, SVRI decreased significantly at 20 min after the termination of CPB compared to baseline (*p* = 0.004). In group B, no significant differences were found in SVRI between successive measurements, while in group A, there was a significant increase two hours after surgery compared to the end of the surgery (*p* = 0.004).

PVR was similar between the two groups 20 min after CPB discontinuation and at the end of surgery. However, two hours after ICU admission, PVR in group A was lower than in group B (*p* = 0.005). Regarding changes over time, a significant decrease in PVR was shown in both groups 20 min after the discontinuation of CPB compared to the baseline (*p* < 0.001 and *p* < 0.001, respectively), and then in group A, there was no further significant change while in group B, there was a significant increase two hours after ICU admission compared to the end of surgery (*p* = 0.001).

PVRI was significantly lower in group A than in group B 20 min after the discontinuation of CPB (*p* = 0.009), at the end of the surgery (*p* = 0.045) and two hours after ICU admission (*p* = 0.003). Regarding the changes over time, a significant decrease in PVRI was shown in both groups 20 min after the discontinuation of CPB compared to the baseline (*p* < 0.001 and *p* < 0.001, respectively), and then in group A, there was no further significant change while in group B, there was a significant increase two hours after surgery compared to the end of surgery (*p* < 0.001).

The changes in LVEF and RV function by TAPSE (mm) by group are presented in Table 6. LVEF was similar in the two groups throughout the follow-up period at all timepoints. No significant changes were found between consecutive LVEF measurements in any of the groups. The degree of LVEF change over time was similar in the two groups. RV function by TAPSE was similar in both groups at baseline and throughout follow-up. There were no significant changes between consecutive measurements in either group, and the degree of change over time was similar in both groups.

## 5. Discussion

According to the results of the present prospective randomized clinical study, both intravenous levosimendan administration (at a dose of 6 mcg/kg) and inhalational milrinone administration (at a dose of 50 mcg/kg) before CPB in cardiac surgical patients with confirmed preoperative PH have been found to be effective in reducing SPAP values after CPB in comparison to the baseline values. Although both agents were effective in reducing SPAP, MPAP, MPAP/MAP, PVR and PVRI, the pulmonary vasodilation afforded by levosimendan seems to be of longer duration and greater magnitude compared to the effect of inhaled milrinone. Furthermore, levosimendan administration was accompanied by a temporary reduction in SVR, which was not clinically translated to a decrease in MAP because of a concomitant increase in CO. For this reason, no significant difference in the incidence of hypotension or the requirement for vasopressor support was found between the patients receiving levosimendan intravenously or milrinone via inhalation. These results imply that using levosimendan prophylactically in cardiac surgical patients with PH may help prevent or attenuate the worsening of preexisting PH and potentially of RVF after CPB. This effect could be due to the vasodilatory and inotropic effects of levosimendan, as well as to its anti-inflammatory properties that could play a role in preventing the exacerbation of PH linked to the systemic inflammatory response triggered by the use of CPB in cardiac surgery.

Both milrinone and levosimendan have inotropic as well as vasodilatory properties that seem ideal for the management of PH. Levosimendan has been extensively used in clinical practice for more than 20 years, primarily for managing acute decompensated chronic heart failure (with most research focusing on its effects on left ventricular function). Currently, in addition to its established use in treating acute and advanced heart failure, levosimendan is being investigated as a potential therapeutic modality for PH [38].

Although the current body of literature on the subject is not extensive, the available data do indicate that the use of levosimendan may have a potentially beneficial role in managing PH and the associated RVF resulting from various etiologies, including PAH, left heart disease and congenital heart disease. In patients with PAH, the ability of levosimendan to inhibit the proliferation of smooth muscle cells in the pulmonary artery and its positive effects on the pulmonary circulation may justify its use in combination with guideline-directed medical therapy. In patients with PH due to left heart disease, the reduction in left ventricular filling pressures, and the improvement of endothelial function and RV systolic function, as well as the improvement of RV pulmonary artery coupling, make levosimendan a promising therapeutic strategy [35,36].

In the perioperative context, levosimendan has been evaluated with conflicting results in various clinical conditions, such as coronary artery bypass graft surgery, valve surgery in patients with a low ejection fraction [39,40,41], heart transplantation [42], and weaning from venoarterial extracorporeal membrane oxygenation [43]. However, evidence supporting the use of levosimendan as an effective therapeutic strategy for the management of PH during cardiac surgery is limited.

Conversely, milrinone, whether administered intravenously or via inhalation, represents a well-established therapeutic option in the treatment of PH during cardiac surgery. Compared to intravenous administration, inhaled milrinone has been shown to reduce pulmonary artery pressure without causing systemic hypotension, a fact also shown in our study, as SVR was not affected by milrinone administration [44]. Moreover, inhaled milrinone administered before CPB prevents the occurrence of endothelial dysfunction after CPB [28] and reduces the severity of PH during cardiac surgery [29]. Therefore, we designed the present randomized trial in a way that the group of patients treated with milrinone could serve as a control group in order to assess the effectiveness of levosimendan in managing PH in cardiac surgical patients and compare its pharmacodynamic characteristics with those of milrinone.

Comparative studies between levosimendan and milrinone in cardiac surgical patients with PH are relatively limited. According to a randomized controlled trial performed by Prachi Nag et al. in 132 pediatric patients with PH undergoing ventricular septal defect closure, both levosimendan and milrinone were effective in reducing pulmonary arterial pressure, but levosimendan was associated with significantly higher inotropic requirements compared to milrinone [45]. Mishra et al. concluded that both milrinone and levosimendan caused a similar decrease in pulmonary arterial pressure and maintained comparable biventricular systolic and diastolic function, but levosimendan resulted in a greater increase in HR, a decrease in SVR, and a greater need for norepinephrine compared with milrinone [46]. Both studies concluded that levosimendan is not clinically better than milrinone. However, in both studies, levosimendan and milrinone were administered intravenously during weaning from CPB, using a bolus dose followed by continuous infusion.

In a previous study from our team, we concluded that the administration of levosimendan prior to CPB in association with vasopressors, if necessary, could be protective against PH exacerbation and subsequent RVF during weaning and after CPB [47]. This study had been designed to compare the effectiveness of different doses of levosimendan, having as an important limitation the absence of a control group.

The present study is the first study comparing the effect of the preemptive administration of levosimendan versus the preemptive administration of milrinone, which is a drug already used in clinical practice in the context of the perioperative management of PH and RVF. Our findings confirmed that inhaled milrinone administered before CPB has a protective effect during cardiac surgery in patients with PH, preventing the exacerbation of PH. Additionally, our results demonstrated that administering levosimendan before CPB in cardiac surgical patients with PH is equally effective to using inhaled milrinone, but with the added benefit of a longer duration of action. According to our findings, the preemptive administration of levosimendan could have a favorable role in preventing the worsening of PH during cardiac surgery and superimposed RVF during the weaning of CPB and in the early postoperative period. It is important to note that these findings are related to the administration of levosimendan before CPB, which was the novelty of our study. This timing of administration could allow for the drug to also exert its anti-inflammatory effects beyond its inotropic and vasodilatory properties, thus preventing the CPB-related exacerbation of preexisting PH and potentially RVF. The conflicting results of the different studies comparing levosimendan and milrinone suggest that a different dose and timing of drug administration can lead to different clinical outcomes.

It is very interesting that apart from the intravenous use of levosimendan, some studies support the administration of the drug via inhalation in order to avoid systemic hypotension that could reduce RV perfusion [34]. According to our findings, the intravenous administration of levosimendan at a dose of 6 mcg/kg does not seem to be associated with clinically significant and sustained systemic arterial hypotension, while it is effective in decreasing pulmonary artery pressure. Perhaps higher doses of levosimendan might be more effective in reducing pulmonary artery pressure and enhance right ventricular function [47]. However, this approach might increase the risk for systemic hypotension, which could necessitate the administration of the drug via inhalation instead.

Our research suggests that levosimendan could indeed have a role in the pharmaceutical treatment of PH during cardiac surgery. However, there is a need for further investigation to determine the optimal dosage and administration method for its effective use in this context.

While this study provides valuable information concerning the management of PH during cardiac surgery, it is important to acknowledge its limitations beyond the small sample size. First, it could be claimed that patients with PH resulting from valvular disease might demonstrate a reduction in pulmonary artery pressure following the surgical correction of the valve pathology, attributed to the alleviation of left-sided obstruction, regardless of any pharmacological intervention. Nevertheless, our investigation revealed that even after surgery, both groups still exhibited a moderate level of PH. This residual PH could be attributed to either structural alterations in the pulmonary vasculature or heightened reactivity of the pulmonary vasculature induced by the systemic inflammatory response. Another limitation is that the evaluation of right ventricular function through echocardiography relied solely on TAPSE. Utilizing multiple echocardiographic methods for assessing RV function could enhance our ability to evaluate its performance comprehensively, enabling us to differentiate between normal and abnormal function more effectively. Additionally, the duration of our follow-up was relatively brief, encompassing the intraoperative period and the initial two hours of ICU stay. Extending the observation period to include the first 24 h postoperatively would provide valuable information in regard to the postoperative period and make the study more comprehensive. However, the comparison of hemodynamic measurements involving intubated patients in mechanical ventilation with hemodynamic data involving spontaneous breathing patients would not be possible.

## 6. Conclusions

In conclusion, based on the findings of this randomized study involving a small number of patients, both levosimendan and milrinone, when administered before CPB in cardiac surgical patients with PH, provide protective effects, as they help reduce pulmonary artery pressure and prevent the worsening of PH and RVF. Levosimendan, in particular, administered intravenously at a single bolus dose of 6 mcg/kg, leads to more significant and longer-lasting pulmonary vasodilation in comparison to the inhalational administration of milrinone. The study results suggest that levosimendan administration before CPB effectively reduces pulmonary artery pressure in patients with preexisting PH undergoing cardiac surgery. This indicates that levosimendan may serve as a valuable tool in optimizing hemodynamics and improving outcomes in this patient population. Further research and clinical trials are necessary in order to provide additional insights into the efficacy of levosimendan and validate its pharmaceutical role in managing PH during cardiac surgery.

## Figures and Tables

**Table 1:** Patient characteristics and group comparisons.

	Group		
	A (Levosimendan)	B (Milrinone)	
	N = 20	N = 20	*p*-Value
Gender			
female, n (%)	14 (70.0)	8 (40.0)	0.057 ++
male, n (%)	6 (30.0)	12 (60.0)	
Age (years), mean (SD)	70.8 (14.1)	68.9 (9.3)	0.618 ‡
BSA (m^2^), mean (SD)	1.8 (0.2)	1.9 (0.3)	0.263 ‡
Type of Surgery, n (%)			0.704 +
ASD	0 (0)	1 (5)	
AVR	4 (20)	3 (15)	
AVR-MVR	2 (10)	5 (25)	
AVR-CABG	0 (0)	1 (5)	
AVR-MVR-TV repair	1 (5)	1 (5)	
AVR-MV repair-TV repair	1 (5)	1 (5)	
AVR-MV repair-TV repair-CABG	1 (5)	0 (0)	
MVR	5 (25)	4 (20)	
MVR-CABG	1 (5)	0 (0)	
MVR-TV repair	2 (10)	0 (0)	
MVR-TV repair-CABG	0 (0)	1 (5)	
MV repair	1 (5)	1 (5)	
MV repair-CABG	2 (10)	1 (5)	
MV repair-TV repair-CABG	0 (0)	1 (5)	
Duration of surgery (min), mean (SD)	320 (42.2)	315 (49.0)	0.731 ‡
CPB duration (min), mean (SD)	135 (51.0)	121 (46.1)	0.368 ‡
ACC duration (min), mean (SD)	101 (19.1)	97 (18.3)	0.503 ‡
Tranfusion of RBC (units), mean (SD)	5 (1.0)	5 (1.0)	>0.999 ‡
Requirement for norepinephrine during weaning from CPB, n (%)	12 (60.0)	12 (60.0)	>0.999 +
Norepinephrine dose mcg/kg/min, median (IQR)	0.04 (0–0.1)	0.06 (0–0.1)	0.644 ‡‡
Requirement for dobutamine during weaning from CPB	11 (55.0)	13 (65.0)	0.519 +
Dobutamine dose mcg/kg/min, median (IQR)	1.5 (0–3.5)	2.3 (0–4.5)	0.339 ‡‡
Requirement for epinephrine during weaning from CPB	1 (5.6)	1 (5.0)	>0.999 ++
Epinephrine dose mcg/kg/min, median (IQR)	0.01 (0.02)	0.01 (0.02)	0.971 ‡‡
ICU stay (h), median (IQR)	96 (46.5–156)	48 (25–101.5)	0.089 ‡‡
Duration of mechanical ventilation in ICU (h), mean (SD)	18 (5.0)	16.5 (4.5)	0.325 ‡
Hospital stay (days), median (IQR)	9 (7–13.5)	7.5 (7–10)	0.175 ‡‡
Requirement for IABP-ECMO, n (%)	1 (5.0)	1 (5.0)	>0.999 ++
Mortality, n (%)	1 (5.0)	1 (5.0)	>0.999 ++

‡ Student’s *t*-test; ‡‡ Mann–Whitney test; + Pearson’s Chi-square test; ++ Fisher’s exact test; BSA: body surface area, ASD: atrial septal defect, AVR: aortic valve replacement, MV repair: mitral valve repair, TV repair: tricuspid valve annuloplasty, MVR: mitral valve replacement, CABG: coronary artery bypass graft, CPB: cardiopulmonary bypass, ACC: aortic cross clamp, RBC: red blood cells, ICU: intensive care unit, IABP: intra-aortic balloon pump, ECMO: extracorporeal membrane oxygenation.

**Table 2:** HR, SAP, DAP, MAP, CVP, SPAP, DPAP, MPAP and MPAP/MAP values at various timepoints, group comparisons.

		Baseline	20′ after Discontinuation of CPB	End of Surgery	2 h Later in ICU
	Group	Mean (SD)	Mean (SD)	Mean (SD)	Mean (SD)
HR (beats/min)	A	72 (21.1)	84.9 (7.9)	83.2 (8.6)	81.1 (11.3)
	B	76.7 (13.5)	85.7 (10.2)	82.7 (8.8)	83.7 (6.9)
	P ^1^	0.407	0.783	0.871	0.395
SAP (mmHg)	A	111.4 (16.7)	109.6 (12.7)	106.9 (12.1)	117.7 (17.2)
	B	117.3 (16.6)	108.7 (11.5)	110.5 (11.4)	113.6 (8.2)
	P ^1^	0.265	0.815	0.338	0.342
DAP (mmHg)	A	61.7 (12.4)	57.1 (10.1)	58.7 (9.4)	61.7 (11.6)
	B	61.8 (10.4)	58.9 (12.3)	58.3 (10.1)	63.3 (7.9)
	P ^1^	0.978	0.625	0.885	0.625
MAP (mmHg)	A	80.4 (12.7)	73.5 (10.1)	73.1 (8.9)	78.5 (13.3)
	B	81.1 (12.6)	76.1 (9.9)	75.9 (10.1)	80.3 (7.3)
	P ^1^	0.862	0.416	0.349	0.610
CVP (mmHg)	A	15.8 (3.6)	15.4 (3.2)	15.9 (3)	14 (2.8)
	B	16.5 (3.3)	14.1 (2.9)	14.3 (1.9)	13.7 (1.7)
	P ^1^	0.499	0.210	0.050	0.734
SPAP (mmHg)	A	54.9 (10.3)	45.4 (8.2)	45.3 (8.1)	46.3 (6.7)
	B	61.3 (12.7)	47.4 (9.5)	47 (8)	52 (7.1)
	P ^1^	0.086	0.481	0.509	0.013
DPAP (mmHg)	A	29.7 (5)	26 (6.6)	26.8 (5)	24.9 (5.1)
	B	34.1 (5.4)	29.1 (6.3)	29.4 (7.1)	33.7 (6.5)
	P ^1^	0.101	0.130	0.181	<0.001
MPAP (mmHg)	A	40.4 (5.5)	32.9 (6.2)	34.6 (5.7)	32.6 (5.7)
	B	44.4 (7.3)	35.8 (6.8)	35.2 (7.9)	40.2 (5.9)
	P ^1^	0.065	0.160	0.802	<0.001
MPAP/MAP	A	0.52 (0.12)	0.44 (0.09)	0.47 (0.1)	0.41 (0.1)
	B	0.56 (0.13)	0.47 (0.1)	0.46 (0.09)	0.5 (0.09)
	P ^1^	0.356	0.363	0.660	0.002

Note. Group A = levosimendan and B = milrinone; ^1^ *p*-value for group effect.

**Table 3:** Baseline values and changes in HR, SAP, DAP, MAP, CVP, SPAP, DPAP, MPAP and MPAP/MAP over time.

		(1)	Change (1)–(2)		Change (2)–(3)		Change (3)–(4)		P ^3^
	Group	Mean (SD)	Mean (SD)	Ρ ^2^	Mean (SD)	Ρ ^2^	Mean (SD)	Ρ ^2^	
HR (beats/min)	A	72 (21.1)	12.9 (20.8)	0.009	−1.7 (7.7)	>0.999	−2.1 (10.8)	>0.999	0.521
	B	76.7 (13.5)	9 (11.4)	0.130	−3 (6.5)	0.433	0.9 (6.3)	>0.999	
	P ^1^	0.407							
SAP (mmHg)	A	111.4 (16.7)	−1.8 (14.7)	>0.999	−2.7 (18.3)	>0.999	10.9 (19)	0.019	0.301
	B	117.3 (16.6)	−8.7 (22.7)	0.301	1.8 (12.3)	>0.999	3.1 (10.5)	>0.999	
	P ^1^	0.265							
DAP (mmHg)	A	61.7 (12.4)	−4.6 (14.1)	>0.999	1.6 (10.5)	>0.999	3 (13.2)	>0.999	0.907
	B	61.8 (10.4)	−3 (15.9)	>0.999	−0.6 (11)	>0.999	5 (9.4)	0.348	
	P ^1^	0.978							
MAP (mmHg)	A	80.4 (12.7)	−6.9 (14.3)	0.337	−0.5 (12.6)	>0.999	5.5 (11.4)	0.130	0.913
	B	81.1 (12.6)	−5 (16.7)	0.977	−0.2 (10)	>0.999	4.3 (8.8)	0.381	
	P ^1^	0.862							
CVP (mmHg)	A	15.8 (3.6)	−0.4 (4.3)	>0.999	0.5 (2)	>0.999	−1.9 (3.1)	0.006	0.088
	B	16.5 (3.3)	−2.4 (2.9)	0.033	0.2 (1.6)	>0.999	−0.6 (1.3)	>0.999	
	P ^1^	0.499							
SPAP (mmHg)	A	54.9 (10.3)	−9.5 (10.4)	0.003	−0.2 (7.7)	>0.999	1 (7.4)	>0.999	0.264
	B	61.3 (12.7)	−13.9 (11.8)	<0.001	−0.5 (4.8)	>0.999	5 (6.3)	0.014	
	P ^1^	0.086							
DPAP (mmHg)	A	29.7 (5)	−3.7 (7.3)	0.132	0.8 (5.5)	>0.999	−1.9 (4.9)	>0.999	0.008
	B	34.1 (5.4)	−5 (6.5)	0.017	0.3 (4.4)	>0.999	4.3 (7.1)	0.022	
	P ^1^	0.101							
MPAP (mmHg)	A	40.4 (5.5)	−7.5 (8)	0.001	1.7 (6.7)	>0.999	−2 (5)	0.808	0.020
	B	44.4 (7.3)	−8.6 (7.3)	<0.001	−0.7 (4.4)	>0.999	5 (6.8)	0.004	
	P ^1^	0.065							
MPAP/MAP	A	0.52 (0.12)	−0.08 (0.13)	0.040	0.03 (0.11)	>0.999	−0.07 (0.09)	0.008	0.017
	B	0.56 (0.13)	−0.09 (0.12)	0.047	−0.01 (0.1)	>0.999	0.04 (0.08)	0.153	
	P ^1^	0.356							

Note. Group A = levosimendan and B = milrinone; (1) Baseline; (2) 20 min after discontinuation of CPB; (3) End of surgery; (4) Two hours later in ICU; ^1^ *p*-value for group effect; ^2^ p-value for the time effect after Bonferroni correction; **^3^** repeated-measures ANOVA *p*-value, regarding the time × group effect.

**Table 4:** PCWP, CO, CI, SV, SVI, SVR, SVRI, PVR and PVRI values at various timepoints: group comparisons.

		Baseline	20′ after Discontinuation of CPB	End of Surgery	2 h Later in ICU
	Group	Mean (SD)	Mean (SD)	Mean (SD)	Mean (SD)
PCWP (mmHg)	A	24.9 (3.6)	23.2 (5.2)	22.9 (4.4)	20.8 (3.4)
	B	24.7 (4)	22 (3.7)	21.6 (2.9)	21.6 (2.3)
	P ^1^	0.869	0.406	0.260	0.362
CO (L/min)	A	3.53 (0.65)	4.15 (0.88)	4.24 (0.63)	4.03 (0.66)
	B	4.08 (0.69)	4.58 (0.9)	4.55 (0.73)	4.42 (0.69)
	P ^1^	0.104	0.135	0.167	0.077
CI (L/min/m^2^)	A	1.98 (0.45)	2.3 (0.51)	2.36 (0.45)	2.25 (0.51)
	B	2.17 (0.41)	2.43 (0.5)	2.42 (0.42)	2.35 (0.36)
	P ^1^	0.174	0.419	0.689	0.458
SV (mL)	A	53.13 (18.11)	49.08 (11.15)	51.24 (12.14)	50.41 (10.73)
	B	55.62 (15.97)	52.46 (11.08)	56.06 (13.34)	52.98 (10.92)
	P ^1^	0.647	0.342	0.240	0.456
SVI (mL/m^2^)	A	29.47 (13.78)	28.16 (8.32)	34.36 (19.4)	28.34 (8.11)
	B	29.87 (9.23)	28.09 (6.12)	29.34 (7.65)	28.22 (5.53)
	P ^1^	0.885	0.978	0.288	0.956
SVR (dyn*s/cm^5^)	A	1480.4 (392.5)	1169.7 (340.8)	1082.2 (230.8)	1343 (412.2)
	B	1287.5 (297.2)	1116.3 (287.6)	1147.1 (269)	1233.3 (222.3)
	P ^1^	0.088	0.595	0.418	0.301
SVRI (dyn*s/cm^5^*m^2^)	A	2713.3 (767.1)	2201.3 (784.5)	1990.4 (549.9)	2445.7 (819.3)
	B	2426.6 (624)	2095.2 (572.2)	2007.7 (641.4)	2260.5 (519.1)
	P ^1^	0.203	0.628	0.927	0.398
PVR (dyn*s/cm^5^)	A	358.9 (108.7)	191.9 (132.7)	197.9 (97.5)	242.6 (124.1)
	B	396.8 (113.5)	246.8 (94.3)	243.5 (82.7)	351.8 (109.6)
	P ^1^	0.288	0.140	0.112	0.005
PVRI (dyn*s/cm^5^*m^2^)	A	650.4 (191.1)	281.2 (231.9)	353.1 (158.3)	435.6 (222.4)
	B	756.8 (245.6)	466.9 (192.7)	458.8 (163.8)	671.2 (239.3)
	P ^1^	0.134	0.009	0.045	0.003

Note. Group A = levosimendan and B = milrinone; ^1^ *p*-value for group effect.

**Table 5:** Baseline values and changes in PCWP, CO, CI, SV, SVI, SVR, SVRI, PVR and PVRI over time.

		(1)	Change (1)–(2)		Change (2)–(3)		Change (3)–(4)		P ^3^
	Group	Mean (SD)	Mean (SD)	Ρ ^2^	Mean (SD)	Ρ ^2^	Mean (SD)	Ρ ^2^	
PCWP (mmHg)	A	24.9 (3.6)	−1.7 (7)	>0.999	−0.3 (3.7)	>0.999	−2.2 (5.2)	0.096	0.375
	B	24.7 (4)	−2.7 (4.4)	0.278	−0.4 (1.8)	>0.999	0.1 (1.5)	>0.999	
	P ^1^	0.869							
CO (L/min)	A	3.53 (0.65)	0.61 (0.77)	0.005	0.1 (0.85)	>0.999	−0.21 (0.52)	0.248	0.669
	B	4.08 (0.69)	0.5 (0.74)	0.055	−0.03 (0.37)	>0.999	−0.13 (0.35)	>0.999	
	P ^1^	0.104							
CI (L/min/m^2^)	A	1.98 (0.45)	0.32 (0.39)	0.006	0.06 (0.46)	>0.999	−0.12 (0.3)	>0.999	0.679
	B	2.17 (0.41)	0.26 (0.4)	0.034	−2.89 (0.22)	>0.999	−0.07 (0.25)	>0.999	
	P ^1^	0.174							
SV (mL)	A	53.13 (18.11)	−4.1 (17.8)	>0.999	2.2 (10.6)	>0.999	−0.8 (9.6)	>0.999	0.861
	B	55.62 (15.97)	−3.2 (11.1)	>0.999	3.6 (7.3)	0.521	−3.1 (5.9)	0.560	
	P ^1^	0.647							
SVI (mL/m^2^)	A	29.47 (13.8)	−1.2 (10.4)	>0.999	6.2 (19.6)	0.362	−6 (18.1)	0.280	0.147
	B	29.87 (9.23)	−1.8 (6.5)	>0.999	1.2 (5.1)	>0.999	−1.1 (3.9)	>0.999	
	P ^1^	0.885							
SVR (dyn*s/cm^5^)	A	1480.4 (392.5)	−310.8 (305.1)	0.001	−87.5 (382.7)	>0.999	260.8 (309.8)	0.001	0.095
	B	1287.5 (297.2)	−171.3 (346.8)	0.146	30.8 (247.2)	>0.999	86.2 (235.1)	>0.999	
	P ^1^	0.088							
SVRI (dyn*s/cm^5^*m^2^)	A	2713.3 (767.1)	−512 (569.3)	0.004	−211 (854.2)	>0.999	455.3 (528.7)	0.004	0.508
	B	2426.6 (624)	−331.3 (656.6)	0.125	−87.5 (563.3)	>0.999	252.8 (578.1)	0.290	
	P ^1^	0.203							
PVR (dyn*s/cm^5^)	A	358.9 (108.7)	−167 (139.1)	<0.001	6 (110.3)	>0.999	44.7 (112.9)	0.495	0.235
	B	396.8 (113.5)	−150 (125.2)	<0.001	−3.2 (56.1)	>0.999	108.2 (111)	0.001	
	P ^1^	0.288							
PVRI (dyn*s/cm^5^*m^2^)	A	650.4 (191.1)	−369.2 (228.9)	<0.001	71.9 (237.2)	0.528	82.5 (202.3)	0.506	0.207
	B	756.8 (245.6)	−290 (245.3)	<0.001	−8.1 (105.3)	>0.999	212.5 (214.8)	<0.001	
	P ^1^	0.134							

Note. Group A = levosimendan and B = milrinone; (1) Baseline; (2) 20 min after discontinuation of CPB; (3) End of surgery; (4) Two hours later in ICU; ^1^ *p*-value for the group effect; ^2^ *p*-value for the time effect after Bonferroni correction; **^3^** repeated-measures ANOVA *p*-value, regarding the time × group effect.

**Table 6:** Changes in LVEF (%) and TAPSE (mm) by group.

		Baseline (1)	End of Surgery (2)	2 h Later (3)	Change (1)–(2)		Change (2)–(3)		P ^3^
	Group	Mean (SD)	Mean (SD)	Mean (SD)	Mean (SD)	Ρ ^2^	Mean (SD)	Ρ ^2^	
LVEF (%)	A	47.3 (7)	48.3 (6.1)	48.3 (7.1)	1 (4.17)	0.886	0 (6.69)	>0.999	0.921
	B	51.3 (6.9)	51.8 (4.5)	52.3 (5.1)	0.5 (4.26)	>0.999	0.5 (3.59)	>0.999	
	P ^1^	0.075	0.059	0.060					
TAPSE (mm)	A	15.5 (2.5)	15.8 (3)	15.7 (2.2)	0.3 (2.41)	>0.999	−0.1 (2.02)	>0.999	0.950
	B	15.6 (1.5)	15.9 (1.1)	15.6 (1.6)	0.3 (0.73)	>0.999	−0.25 (1.12)	>0.999	
	P ^1^	0.879	0.890	0.934					

Note. Group A = levosimendan and B = milrinone, ^1^ *p*-value for group effect; ^2^ *p*-value for the time effect after Bonferroni correction; **^3^** repeated-measures ANOVA *p*-value, regarding the time × group effect.

## Data Availability

The datasets generated and/or analyzed during the current study are available from the corresponding author on reasonable request.
